# Exploring the Association Between Gut Microbiota and Infertility in Women with Multiple Implantation Failures: An Exploratory Study

**DOI:** 10.3390/microorganisms14061334

**Published:** 2026-06-14

**Authors:** Giada La Placa, Gemma Fabozzi, Barbara Pala, Daniele Peluso, Danilo Cimadomo, Alberto Vaiarelli, Paola Gualtieri, Laura Di Renzo

**Affiliations:** 1 PhD School of Applied Medical-Surgical Sciences, Tor Vergata University of Rome, 00133 Rome, Italy; laplacagiada@gmail.com (G.L.P.); barbara.pala93@gmail.com (B.P.); daniele.peluso@uniroma2.it (D.P.); 2 School of Specialization in Food Science, Tor Vergata University of Rome, 00133 Rome, Italy; gemmafabozzi@hotmail.it; 3 Department of Biomedicine and Prevention, University of Tor Vergata, 00133 Rome, Italy; 4 IVIRMA Global Research Alliance, IVIRMA Italia, Reproductive Health Unit, 00197 Rome, Italy; 5 Unit of Cardiology, Istituto Dermatopatico Dell’immacolata (IDI)—IRCCS, Via Monti di Creta, 00167 Rome, Italy; 6 Department of Biology, Centro di Bioinformatica Molecolare, Tor Vergata University of Rome, 00133 Rome, Italy; 7 Department of Biology and Biotechnology “Lazzaro Spallanzani”, University of Pavia, 27100 Pavia, Italy; danilo.cimadomo@unipv.it; 8 IVIRMA Global Research Alliance, IVIRMA Italia, Science Research and Education Unit, 00197 Rome, Italy; 9 IVIRMA Global Research Alliance, Genera, Clinica Valle Giulia, 00197 Rome, Italy; alberto.vaiarelli@gmail.com; 10 Section of Food, Clinical Nutrition and Pharmaceutica Science, Department of Biomedicine and Prevention, Tor Vergata University of Rome, 00133 Rome, Italy

**Keywords:** infertile women, implantation failures, gut microbiota composition, comparison

## Abstract

Implantation failure remains a major challenge in IVF, and the contribution of the gut microbiota to implantation success is still poorly defined. We conducted a pilot matched case–control study (February 2023–December 2024) to compare gut microbiota profiles between women with RIF (defined according to ESHRE good practice recommendations) and fertile controls with documented fertility (≥1 prior spontaneous pregnancy). All participants underwent standardized clinical and nutritional assessment of medical history, dietary habits, anthropometry, and body composition. Stool samples were collected for 16S rRNA gene sequencing. In women with RIF, sampling occurred within 1 year after the last failed embryo transfer. Of 45 enrolled women, 41 completed the study (20 RIF and 21 controls; mean age 38.46 ± 4.53 years), with no significant between-group age differences. Women with RIF showed reduced alpha diversity (Shannon *p* = 0.003; inverse Simpson *p* = 0.002) and a distinct community structure versus controls (Bray–Curtis PERMANOVA F = 7.16; R^2^ = 0.16; *p* = 0.001), which remained significant after adjustment for clinical covariates including waist-to-hip ratio (*p* = 0.018). At the phylum level, women with RIF had fewer Firmicutes (52.7% vs. 65.0%; *p* = 0.012) and more Proteobacteria (9.1% vs. 3.6%; *p* < 0.001). These findings support an association between gut dysbiosis and a history of implantation failures and warrant confirmation in larger, longitudinal cohorts.

## 1. Introduction

Infertility, defined by the failure to achieve a pregnancy after 12 months or more of regular unprotected sexual intercourse [1], is a disease that affects 48 million couples and up to 186 million individuals of reproductive age in the world [2]. It has a profound impact on their lives, leading to psychological distress as well as relationship difficulties [1]. Assisted reproduction technologies (ARTs) offer significant opportunities for infertile parents to realize their family goals; however, despite the advantages in reproductive medicine, fewer than half of IVF cycles result in a live birth, with success rates ranging from 10% to 30% [3,4].

Implantation is one of the critical aspects that continue to lack comprehensive understanding. Indeed, even if IVF results in euploid blastocysts for transfer, they fail to implant in 40–50% of cases [5].

Embryo implantation is a multifactorial process that requires optimal embryo competence, a receptive endometrium, and effective communication between the blastocyst and the endometrial lining [6]. Various systemic factors—such as hormonal imbalances, coagulation disorders, and immune dysregulation—have been implicated in implantation failure; however, key aspects of its pathophysiology remain poorly understood [7]. One emerging factor in reproductive success is the role of the microbiota, particularly within the lower reproductive tract. Over the past decade, the composition of the endometrial microbiota has been extensively studied in relation to implantation failure and adverse pregnancy outcomes [8], along with potential mechanisms linking microbial imbalance to early pregnancy loss. A dysbiotic endometrial microbiota appears to be capable of modulating crucial inflammatory pathways necessary for successful embryo implantation and the progression of pregnancy [9]. Bacterial transmission routes between the uterine microbiota and distant body sites have been identified, including the established ascension of bacteria through the cervix from the vagina, as well as hematogenous spread from the gut microbiota [10,11]. Recently, the vaginal microbiota has been extensively studied in relation to both reproductive [12,13,14,15] and obstetric outcomes [16,17]. In contrast, the gut microbiota remains relatively underexplored in this context.

The human gut microbiota constitutes the largest microbial community in the body [18]. These microbial communities exist in symbiosis with the host, contributing to overall homeostasis by supporting nutrient absorption, protecting against pathogenic microorganisms, and regulating key physiological processes, including the development and modulation of the immune system [19].

The composition and stability of the gut microbiota are influenced by multiple factors, including diet, age, lifestyle, medication use (particularly use of antibiotics), environmental exposures, and host genetics [20]. When exposed to a perturbation, the composition of the microbiota may change in terms of its quality, quantity, and functionality, switching from a eubiotic to a dysbiotic state [21] characterized by a reduction in microbial diversity, depletion of beneficial commensal bacteria, overgrowth of potentially pathogenic microorganisms, and alterations in microbial metabolic activity [20].

When microbial communities are resilient, eubiosis can be restored; conversely, if an unhealthy dysbiotic state persists over time, it may contribute to the development of microbiota-associated diseases including cardiovascular diseases (CVDs), cancer, and respiratory conditions [22].

More recent research suggests a potential link between specific gut bacteria and disorders of the female reproductive tract that can impair fertility [23], including ovarian cancer [24], endometrial cancer [25], polycystic ovary syndrome (PCOS) [26,27,28], endometriosis [29,30,31], and uterine fibroids (UFs) [32,33].

Notably, the GM can also influence sex hormone levels. Certain gut bacteria, collectively known as the estrobolome, possess β-glucuronidase (GUSB) activity, which plays a crucial role in estrogen metabolism through modulation of the enterohepatic circulation. Consequently, chronic dysbiosis may contribute to hormonal imbalances, thereby impacting fertility [23].

Considering these findings, this study aims to investigate whether differences exist in the GM composition between infertile patients with RIF defined according to the ESHRE 2023 criteria [7] and matched fertile controls, to better understand the role of the microbiota in implantation failure and its potential as a therapeutic target.

## 2. Materials and Methods

### 2.1. Study Design

The pilot matched case–control study was conducted from February 2023 to December 2024 at the Clinical Nutrition and Nutrigenomics Department of Food Science, Clinical Nutrition, and Pharmacology of the Tor Vergata University of Rome. All participants recruited into the study read and signed the informed consent form, in accordance with the 1975 Declaration of Helsinki, as revised in 2013. The study protocol has been approved by the Ethics Committee of the Calabria Region Center Area Section (Register Protocol No. 97, 20 April 2023).

To be eligible for the study, women had to be between 18 and 46 years of age and had to either have successfully delivered ≥1 live birth ≥6 months prior (control group) or be an infertile patient with a history of idiopathic implantation failures after IVF as defined according to the ESHRE 2023 good practice recommendations document [7], namely, a history of ≥2 embryo transfers that did not result in a positive pregnancy test with a frequency that warrants further investigation and/or intervention (>60%). The test was offered free of charge as part of a research study. Additional exclusion criteria included maternal age < 18 or >46 years, previous hysterectomy, current breastfeeding, HIV/AIDS infection, ongoing cancer treatment, and the use of broad-spectrum antibiotic therapy within 30 days prior to stool sample collection and microbiota analysis. The study recruited a total of 45 patients with different environmental backgrounds, eating habits, lifestyles, body compositions, and BMI scores. All enrolled subjects underwent a comprehensive assessment, which included assessment of their medical history, lifestyle habits, dietary patterns, nutritional status, body composition, and gut microbiota composition.

### 2.2. Data Collection

All patients were evaluated by complete medical, nutritional, and dietary examinations, which included anthropometric measurements (weight, height, circumferences). Stool samples were collected to analyze the compositional profile of the GM.

### 2.3. Anthropometry

The weight and height of the participants were assessed using a stadiometer and calibrated scales (Invernizzi, Rome, Italy), with participants standing upright and wearing only underwear. Height was recorded to the nearest 0.1 cm and weight to the nearest 0.1 kg [34]. BMI was determined using the standard formula, which divides body weight in kilograms by the square of height in meters [35]. According to the classification provided by the World Health Organization (WHO) [36], BMI values can be interpreted as follows: underweight is defined as a BMI below 18.5 kg/m^2^, normal weight as 18.5–24.9 kg/m^2^, overweight as 25.0–29.9 kg/m^2^, and obesity as a BMI equal to or greater than 30.0 kg/m^2^. Waist and hip circumferences were assessed using a flexible, non-elastic measuring tape as part of the detailed anthropometric evaluation [36]. The waist-to-height ratio (WHR) was calculated using a cutoff value of 0.5 [37].

### 2.4. Gut Microbiota Composition Analysis

Biological samples were obtained from all participants and processed for microbiota profiling. In the RIF group, stool samples were obtained within 1 year from the last failed transfer.

Each participant was provided with a standardized stool sampling kit and received both written and oral instructions on the correct procedures for sample collection, storage, and transport. Stool samples were self-collected at home after evacuation, with participants taking care to avoid contact with urine, toilet water, or other potential contaminants. The samples were subsequently delivered to the clinic following the provided protocol and then transported under controlled conditions to Wellmicro^®^ (Via Antonio Canova 30, 40138 Bologna, Italy) for gut microbiota profiling.

Fecal DNA was extracted and subjected to high-throughput sequencing for taxonomic classification and community structure analysis. The analysis of the gut microbiota was carried out by 16S rRNA sequencing. Sequencing was performed via Next-Generation Sequencing, characterized by massive parallel sequencing, through the ThermoFisher Scientific Ion S5 platform. The results obtained from the sequencing were then compared with large bacterial databases, including Curated Greengenes v135 [38] and the Curated MicroSEQ(R)16S Reference Library [39]. Sequencing data analysis and taxonomic classification were conducted through the QIIME suite [40] and lonReporter software (ThermoFisher Scientific, Waltham, MA, USA). Bacteria were classified according to four taxonomic levels: phylum, family, genus, and species.

Sequence homology was used to identify bacterial groups at different taxonomic levels. Sequence homology of at least 99% allowed for species-level identification, while sequence homology between 97% and 99% allowed for gender-level identification. Sequences with a sequence homology of less than 97% have been identified at the family level. The composition of the gut microbiota was reported as the relative abundance, expressed as a percentage of the total, for each bacterial group. Bacterial groups were defined according to their taxonomic classification at the phylum, family, genus, and species levels. Microbial community profiling was performed using taxonomic annotation of operational taxonomic units (OTUs).

### 2.5. Statistical Analysis

Statistical and bioinformatics analyses were performed in the R v4.4/Bioconductor v3.19 environment, using the R packages phyloseq v1.48.0, microbiome v1.26.0, ANCOMBC v2.6.0, vegan v2.6-6.1, and shiny v1.8.1.1. Descriptive statistics were utilized to summarize the demographic and anthropometric parameters of the participants. Continuous variables are reported as mean ± Standard Deviation (SD), whereas categorical variables are presented as frequency and percentage. Baseline characteristics of the fertile and RIF groups were compared using PERMANOVA with the adonis2 function, implemented in the Vegan R package [41], based on a Euclidean distance matrix.

No formal a priori power analysis or sample size estimation was performed. Given the pilot and exploratory design of the study, the sample size was based on the number of eligible participants recruited during the study period who completed stool sample collection and microbiota profiling. Therefore, the statistical analyses were intended to identify preliminary associations and generate hypotheses for future larger studies rather than to provide definitive confirmatory evidence.

#### Diversity and Multivariate Analyses

Microbial community structure was evaluated using both alpha and beta diversity metrics. Alpha diversity was assessed using the Shannon and inverse Simpson indices, while beta diversity was quantified using Bray–Curtis dissimilarity and visualized through Principal Coordinates Analysis (PCoA). The Bray–Curtis metric was selected because 16S rRNA relative abundance data are compositional, making it a more appropriate measure of ecological distance than Euclidean-based approaches. PCoA was applied as an unsupervised method; group labels were not used during ordination and were added to plots post hoc for visualization only.

Associations between fertility status and alpha diversity were assessed using the Wilcoxon–Mann–Whitney test. Beta diversity comparisons were performed with PERMANOVA on Bray–Curtis dissimilarity matrices.

Differential abundance analysis of microbial taxa was conducted using the Analysis of Compositions of Microbiomes with Bias Correction (ANCOM-BC) method [42]. This approach accounts for differences in sampling fractions by applying a log-linear regression model with estimated sampling fractions as offset terms. The ANCOM-BC package in R was used to identify taxa that were differentially abundant between fertile and RIF groups. Results are reported as log fold changes, standard errors, test statistics, raw and adjusted *p*-values, and taxa identified as significantly differentially abundant at both the family and genus levels. Adjusted *p* < 0.05 was considered statistically significant.

## 3. Results

### 3.1. Study Participants and Baseline Characteristics

In the first phase, 45 participants were enrolled in the study based on the inclusion criteria. After the initial baseline assessment, four participants withdrew because they were unwilling to undergo genetic analysis of their microbiota.

Therefore, 41 participants (20 women with RIF [48.8%] and 21 fertile women [51.2%]) with a mean age of 38.46 ± 4.53 years completed the study. The general characteristics of the study population are described in Table 1.

### 3.2. Preliminary Analysis

Prior to conducting the microbiota analysis, we examined baseline differences among participants to identify potential confounding variables for subsequent analyses. We applied permutational multivariate analysis of variance (PERMANOVA), setting statistical significance at *p* < 0.05.

In SF1, the PERMANOVA yielded a pseudo-F statistic, which reflects the ratio of between-group to within-group variance.

A higher pseudo-F value indicates greater dissimilarity between groups relative to intra-group variability, thus implying a stronger group effect.

As expected, the variables explaining the greatest variance were number of full-term pregnancies and number of pregnancies (pseudo-F = 107.9 and 79.8, respectively). Waist-to-hip ratio (WHR) also showed a significant association with the variance among participants (pseudo-F = 6.65, *p* = 0.011; R^2^ = 0.146), although the effect size was markedly smaller than that of the reproductive variables. This indicates that, while WHR contributes less to between-group dissimilarity, it may still act as a confounding factor. Accordingly, WHR was included as a covariate in subsequent PERMANOVAs of beta diversity to adjust for its potential influence. This preliminary step allowed us to proceed with the microbiota analysis confident that any observed differences would be attributable to microbiota composition rather than baseline confounding factors.

### 3.3. Microbiota Composition

**Figure 1:**
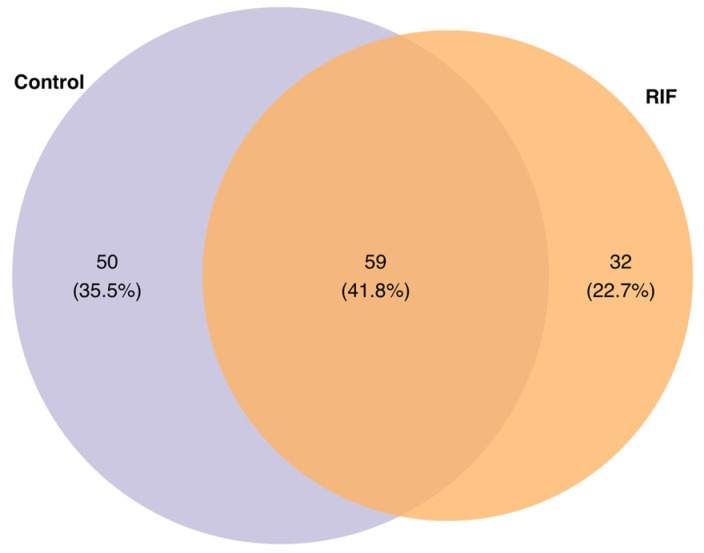
Venn diagram showing the distribution of operational taxonomic units (OTUs) between control and recurrent implantation failure (RIF) groups. The diagram illustrates the number of OTUs shared between groups and those uniquely detected in each group. A total of 141 OTUs were identified across all samples, of which 59 (41.8%) were common to both groups, 50 (35.5%) were unique to the control group, and 32 (22.7%) were exclusive to the RIF group.

The Venn diagram analysis identified a total of 141 OTUs: 59 were shared between groups, 50 were unique to the fertile group, and 32 were exclusive to the RIF group (Figure 1).

**Figure 2:**
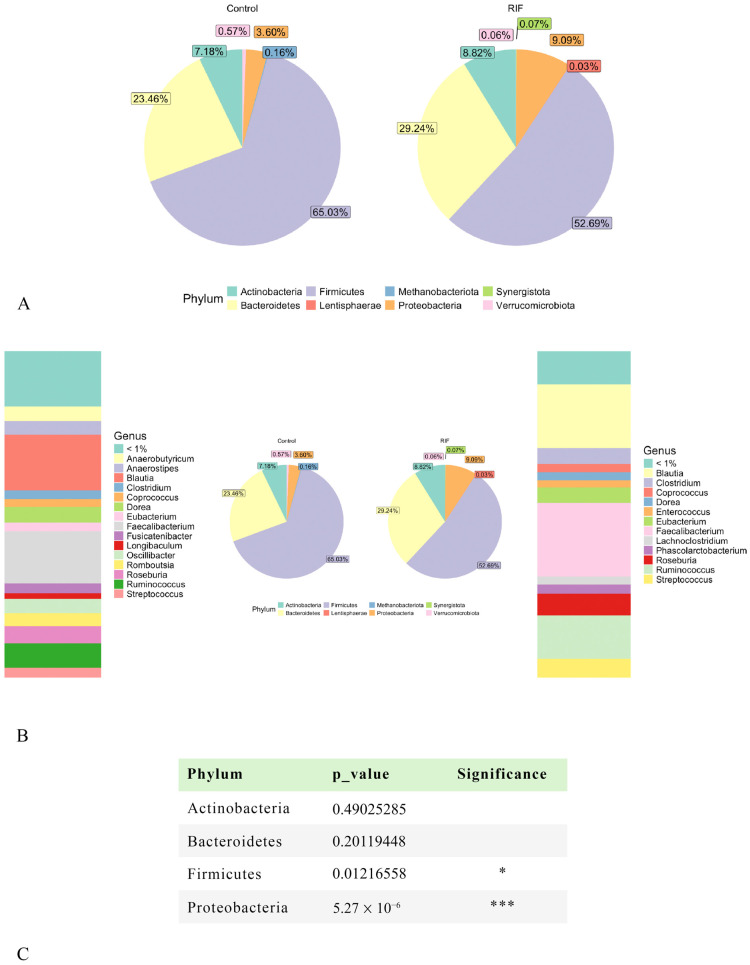
Comparison of bacterial community composition between fertile controls and the RIF group at phylum and genus levels. (**A**) Pie charts showing relative abundance (%) of bacterial phyla in each group. (**B**) Stacked bar plots showing genus-level composition within the Firmicutes phylum. Each color represents a genus; the *y*-axis indicates relative abundance (%). (**C**) Results of Wilcoxon–Mann–Whitney tests comparing phylum-level relative abundances between groups. Statistical significance is indicated as follows: *p* < 0.05 (*), *p* < 0.001 (***).

At the phylum level, the Firmicutes phylum was significantly more abundant in the fertile group (65.03% vs. 52.69%; *p* = 0.012), whereas Proteobacteria was increased in the RIF group (9.09% vs. 3.59%; *p* < 0.001) (Figure 2A,C). Within the Firmicutes phylum, at the genus level, marked differences were evident between the two groups (Figure 2B). In the control group, *Faecalibacterium*, *Roseburia*, *Ruminococcus*, and *Eubacterium* comprised a larger proportion of Firmicutes than in the RIF group, which displayed both a reduced overall abundance of Firmicutes and a distinct shift in genus composition.

### 3.4. Alpha and Beta Diversity Analysis

**Figure 3:**
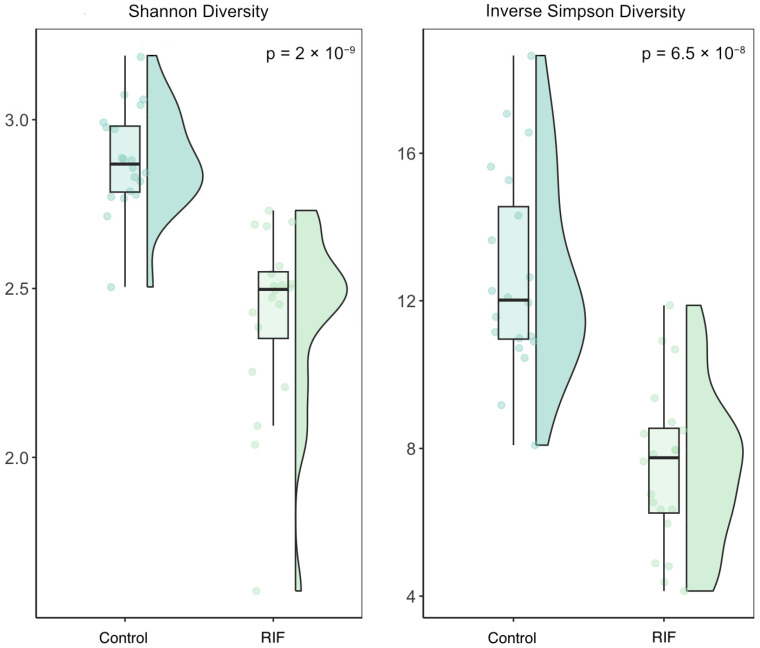
Alpha diversity comparison between fertile controls and the RIF group. Alpha diversity was assessed using the Shannon index and inverse Simpson index, which reflect microbial community richness and evenness. Both indices were significantly lower in the RIF group. Group differences were tested using the Wilcoxon rank-sum test (Shannon: *p* = 0.003; inverse Simpson: *p* = 0.002).

The Shannon index (*p* = 0.003) and inverse Simpson index (*p* = 0.002) were significantly lower in the RIF group (Figure 3), indicating reduced richness and evenness.

**Figure 4:**
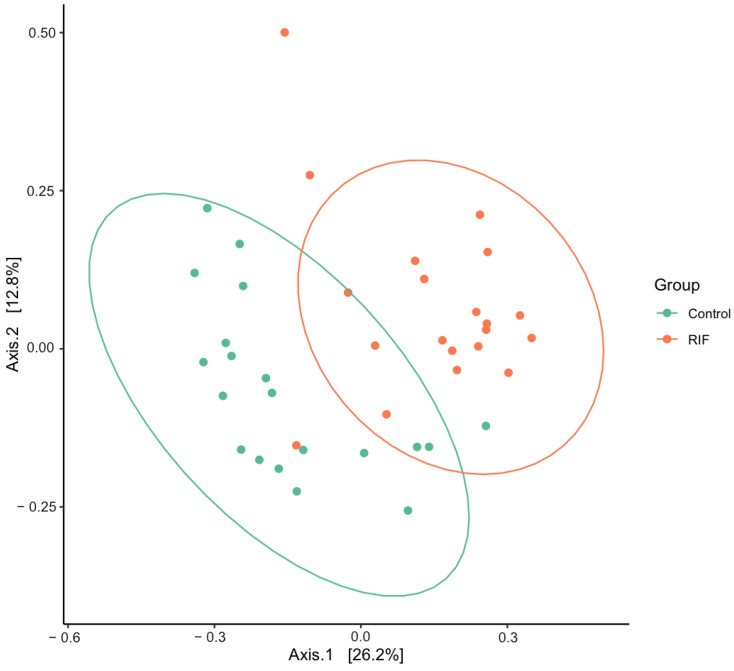
Principal coordinates analysis (PCoA) of gut microbial community structure based on Bray–Curtis dissimilarity. PCoA (principal coordinates analysis) was performed using the Bray–Curtis distance from taxon relative abundance data. Each point represents one sample, positioned according to overall community composition. Group separation was tested using PERMANOVA (F = 7.16; R^2^ = 0.16; *p* = 0.001), with homogeneity of dispersion confirmed (*p* > 0.05). A covariate-adjusted PERMANOVA including clinical metadata confirmed that group (control vs. RIF) remained a significant contributor to variance (F = 2.423; R^2^ = 0.0507; *p* = 0.018).

PCoA based on Bray–Curtis distances revealed a clear separation between the clusters of the two groups (PERMANOVA: F = 7.16; R^2^ = 0.16; *p* = 0.001), with homogeneity of dispersion confirmed (*p* > 0.05) (Figure 4).

To account for potential confounding factors identified in the baseline characteristics analysis (Appendix A), we repeated the PERMANOVA including clinical metadata variables as covariates. This multivariate analysis confirmed that the group variable (control vs. RIF) remained the strongest determinant of variance in gut microbial community structure (R^2^ = 0.0507, F = 2.423, *p* = 0.018).

**Figure 5:**
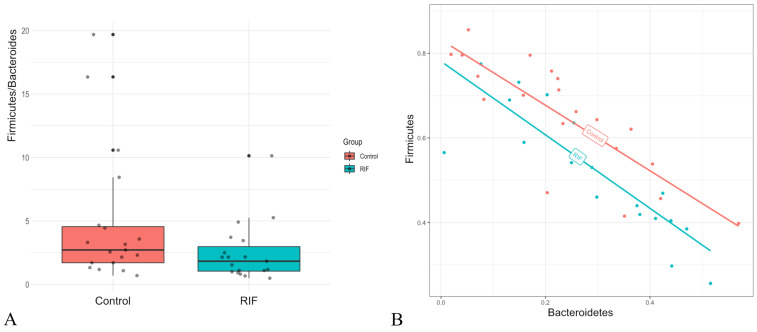
Comparison of the Firmicutes/Bacteroidetes (F/B) ratio between control and RIF groups. (**A**) Boxplot showing F/B ratios per group. The *y*-axis represents the Firmicutes-to-Bacteroidetes ratio. (**B**) Scatterplot showing the relative abundance of Firmicutes and Bacteroidetes for individual samples. Each dot represents one individual sample, and colors indicate the study groups. Regression lines show group-specific trends. No statistically significant difference was observed between groups (Wilcoxon rank-sum test, *p* = 0.091). F/B, Firmicutes-to-Bacteroidetes ratio.

The Firmicutes/Bacteroidetes (F/B) ratio did not show statistically significant differences between groups (*p* = 0.091) (Figure 5).

**Figure 6:**
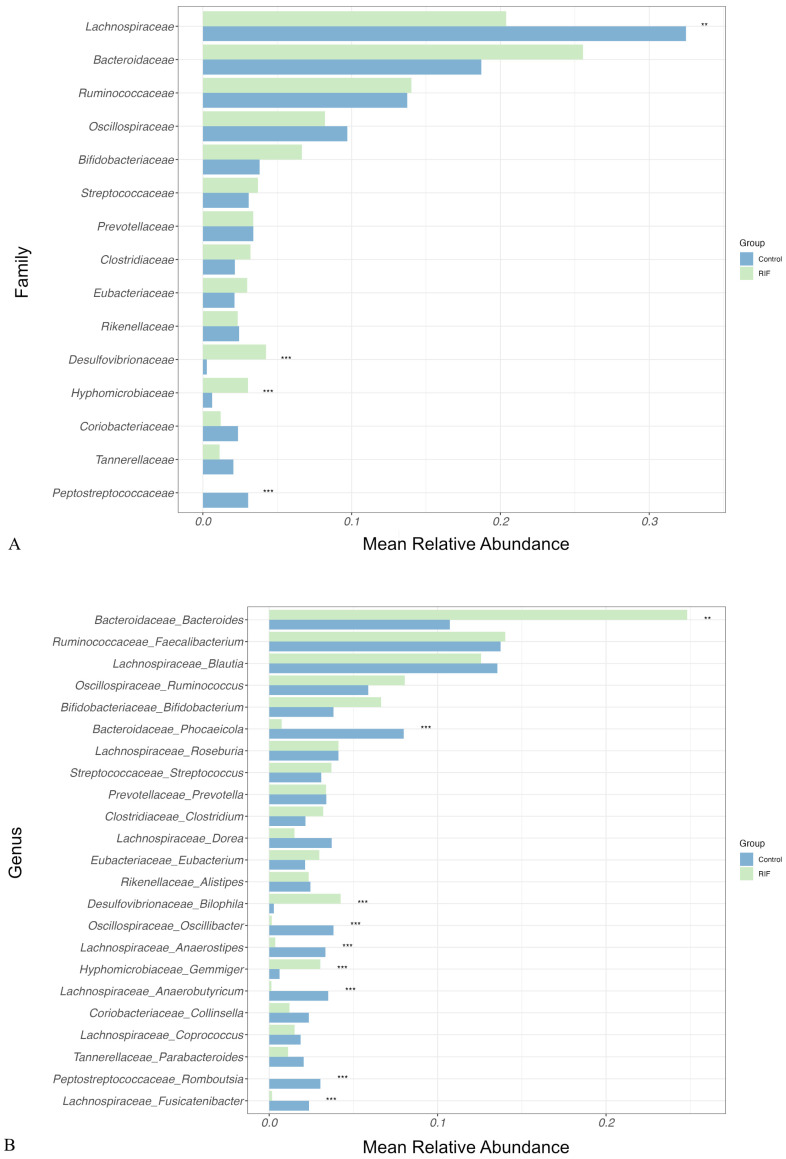
Mean relative abundance of bacterial taxa at the family and genus levels (RIF vs. controls). Rare taxa with total relative abundance < 1% across all samples were excluded. Remaining abundances were aggregated by sample, taxon (family or genus), and group. (**A**) Bar plot of mean relative abundance at the family level. (**B**) Bar plot at the genus level. Differences between groups were assessed using the Wilcoxon–Mann–Whitney test. Asterisks indicate statistically significant differences between groups for the corresponding taxa: *p* < 0.01 (**), *p* < 0.001 (***).

At both the family and genus levels, rare taxa with a total relative abundance of <1% across all samples were excluded. Abundances were then aggregated by sample, taxon (family or genus), and group. For each group, the mean relative abundance of each taxon was calculated. Differences in taxon abundance between fertile and infertile individuals were assessed using the Wilcoxon–Mann–Whitney test (Figure 6).

### 3.5. Differential Abundance

**Figure 7:**
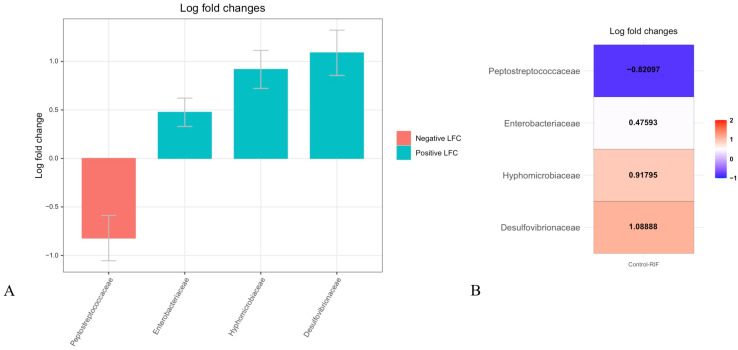
Differential abundance of bacterial genera between fertile controls and the RIF group identified by ANCOM-BC. ANCOM-BC (Analysis of Composition of Microbiomes with Bias Correction) was used to identify genera with significant differences (*p*adj < 0.05). (**A**) Bar plot of log fold change (logFC) estimates for each genus. A positive logFC indicates enrichment in the RIF group; a negative logFC indicates depletion. Error bars represent standard errors. (**B**) Heatmap of logFC values for the same genera; color intensity reflects magnitude and direction of differential abundance.

ANCOM-BC revealed an enrichment of pro-inflammatory families such as *Desulfovibrionaceae*, *Hyphomicrobiaceae*, and *Enterobacteriaceae* in the RIF group (logFC > 1; *p_adj_* < 0.05), along with a depletion of *Lachnospiraceae* and *Peptostreptococcaceae* (Figure 7).

**Figure 8:**
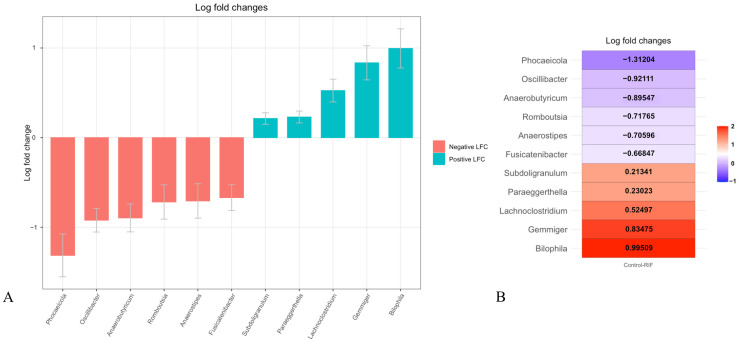
Differential abundance of bacterial families between fertile controls and RIF group identified by ANCOM-BC. ANCOM-BC was used to identify families with significant differences (*p*adj < 0.05). (**A**) Bar plot of logFC estimates for each family. A positive logFC indicates enrichment in the RIF group; a negative logFC indicates depletion. Error bars represent standard errors. (**B**) Heatmap of logFC values for the same families; color intensity reflects the magnitude and direction of differential abundance.

At the genus level, *Bilophila* and *Gemmiger* were found to be overabundant, while *Phocaeicola*, *Anaerobutyricum*, *Oscillibacter*, *Anaerostipes*, *Romboutsia*, and *Fusicatenibacter*—known short-chain fatty acid (SCFA) producers—were significantly reduced (Figure 8).

**Figure 9:**
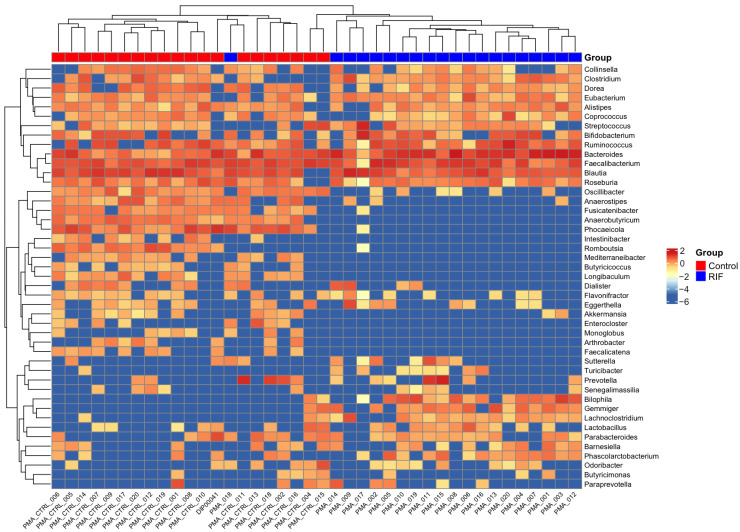
Heatmap of gut microbial genus-level composition in fertile controls and the RIF group. Genera present in at least 20% of samples were included to reduce dimensionality and minimize noise. Relative abundances were log-transformed to compress the dynamic range and reduce the influence of highly dominant taxa. Hierarchical clustering was applied to both samples and genera. Each cell represents the log-transformed relative abundance of a genus in a sample; color intensity indicates higher or lower abundance.

A community heatmap (Figure 9) was used to display compositional patterns at the genus level. To reduce dimensionality and noise, only genera present in at least 20% of the samples were selected. Abundances were transformed to a logarithmic scale to compress the dynamic range and avoid biases due to highly dominant taxa.

## 4. Discussion

This study figures amongst the first comparative analyses of GM composition considering fertile women and infertile patients with RIF, as defined according to the recent ESHRE criteria, showing significant differences across multiple taxonomic levels. Women with RIF and controls shared 41.8% of OTUs, suggesting a baseline microbiota composition (Figure 1). However, the presence of unique taxa in each group, particularly in the control group (35.5% vs. 22.7%), reflects a reduction in microbial diversity together with a shift in microbial structure in infertile women with a history of implantation failures. The analysis of α diversity revealed significantly reduced microbial richness and evenness in the RIF group compared to fertile controls (Figure 3). Both the Shannon diversity index and the inverse Simpson index showed markedly lower values in the RIF group. These indices reflect not only the number of microbial taxa present (richness), but also the relative distribution of their abundances (evenness), providing a measure of community complexity. Notably, α diversity is a critical determinant of gut ecosystem health, with high diversity being associated with better mucosal integrity, metabolic flexibility, and immune regulation [43]. Conversely, a reduced α diversity has been associated with a range of pathological or suboptimal conditions, including use of antibiotics [44], inflammatory diseases such as inflammatory bowel disease (IBD) and obesity [45], metabolic disorders such as type 2 diabetes, insulin resistance, and metabolic syndrome [46], poor dietary habits such as low-fiber, Western diets [47], and psychological stress and depression [48]. Its reduction in the RIF group is consistent with prior studies reporting lower GM diversity in women affected by polycystic ovary syndrome (PCOS) [49], endometriosis [50], and idiopathic infertility [51], highlighting a potential link between gut dysbiosis and impaired reproductive function through the induction of systemic inflammation, metabolic disturbances, or hormonal imbalances.

Analysis of β diversity, not addressed in previous studies, revealed a clear separation between the gut microbiota profiles of controls and women with RIF (Figure 3). The two groups formed distinct clusters, indicating that the overall composition of the microbial communities differed significantly between them. This may lead to the speculation that women with RIF possess not just specific taxonomic imbalances, but a broader ecological reorganization of the gut microbiome. Indeed, the difference in β diversity reflects that women with RIF have a qualitatively different community structure.

With regard to phylum abundance, shown in Figure 2A, the RIF group exhibited two important significant differences: lower relative abundance of Firmicutes (52.7%) compared to fertile controls (65%) (*p* = 0.012) and a significant increase in Proteobacteria (9.1%) with respect to fertile controls (3.6%) (*p* < 0.000001). A difference in terms of Firmicutes between the two groups was also observed by Manzoor et al. (2021), whose results showed reduced Firmicutes representation in women with RIF [52]. Interestingly, this difference is not just associated with a quantitative reduction of bacteria, but with a qualitative alteration as well (Figure 2B). Indeed, the Firmicutes phylum in fertile patients is characterized by a greater presence of genera with recognized anti-inflammatory and barrier-supportive functions, such as *Faecalibacterium*, *Roseburia*, *Ruminococcus*, and *Eubacterium*—all known producers of short-chain fatty acids (SCFAs), particularly butyrate, contributing to epithelial integrity, immune tolerance, and metabolic regulation [53]. In contrast, the GM of women with RIF shows a relative reduction or absence of these key butyrate-producing Firmicutes and a higher proportion of genera such as *Streptococcus*, *Lachnoclostridium*, and *Enterococcus*, which have been associated with pro-inflammatory profiles [54], autoimmune diseases [55,56], cancer [57], and other pro-inflammatory diseases [58,59].

With regard to the increased abundance of *Proteobacteria* in the RIF group, this finding aligns with those of Patel et al. (2022), who demonstrated an increased abundance of Proteobacteria in women with RIF [51]. This phylum, widely recognized as a hallmark of microbial dysbiosis and epithelial dysfunction, encompasses a broad group of Gram-negative bacteria, including genera such as *Escherichia*, *Klebsiella*, and *Enterobacter*, known for their potential pathogenicity and ability to elicit pro-inflammatory responses through the production of lipopolysaccharide (LPS) [60]. LPS, a potent endotoxin present in the outer membrane of these bacteria, activates toll-like receptor 4 (TLR4) on intestinal epithelial and immune cells, triggering low-grade systemic inflammation [61]. Furthermore, an overrepresentation of Proteobacteria is frequently observed in conditions characterized by immune dysregulation and metabolic disturbance—including obesity, type 2 diabetes, IBD, PCOS, and endometriosis [62].

Regarding the Firmicutes/Bacteroidetes (F/B) ratio, a widely recognized marker of GM homeostasis and metabolic status, we observed a lower ratio in women with RIF (Figure 5), as also reported previously by other authors [52]. However, no statistically significant difference was observed, which might be explained by the difference in richness between the two populations. In fact, the uneven biodiversity in the two groups likely masked group-level differences in the F/B ratio, making it less reliable as a standalone marker. At the family level, instead, our analysis revealed several statistically significant differences in bacterial composition between fertile women and women with RIF, highlighting specific microbial signatures potentially associated with reproductive health (Figure 6A).

Two main families were significantly less abundant in women with RIF: *Lachnospiraceae* and *Peptostreptococcaceae*. These families include multiple genera able to ferment dietary fiber [63] and produce beneficial short-chain fatty acids (SCFAs) like butyrate, which promote healthy gut and immune function and exert anti-inflammatory effects [64,65]. Indeed, their depletion may contribute to a pro-inflammatory gut environment, such as in the case of irritable bowel syndrome (IBS), aligning with the findings of Bellver et al. (2024) and Pozuelo et al. (2015) [66,67].

On the contrary, the families *Desulfovibrionaceae* and *Hyphomicrobiaceae* were significantly more abundant in women with RIF. These families are often associated with pro-inflammatory or potentially pathogenic activity. For instance, *Desulfovibrionaceae* are sulfate-reducing bacteria that produce hydrogen sulfide, a compound implicated in mucosal damage and intestinal permeability [68]. Moreover, an increase in *Desulfovibrionaceae* has been described in metabolic syndrome, obesity, and ulcerative colitis, and may similarly contribute to an inflammatory milieu in the context of infertility [69]. Some bacteria within the *Hyphomicrobiaceae* family, consisting of Gram-negative bacteria, have been identified in the blood of individuals with rheumatoid arthritis [70]. Furthermore, this family has recently been identified as part of pro-inflammatory gut microbiota profiles in women with idiopathic infertility and poor reproductive outcomes [51].

To quantify the magnitude of the observed taxonomic differences, a log fold change analysis at the family level was performed, where only taxa with statistically significant differences were retained (Figure 7). The most positive fold changes, indicating higher abundance in the RIF group, were observed for *Desulfovibrionaceae*, *Hyphomicrobiaceae*, and *Enterobacteriaceae*, all of which are known for their pro-inflammatory or opportunistic potential. The latter family is especially interesting, as it encompasses numerous opportunistic pathogens, including *Escherichia* and *Klebsiella*, and is commonly elevated in conditions of gut dysbiosis. In reproductive settings, this family has been implicated in endometriosis, impaired implantation, and poor ART outcomes [71,72]. In contrast, *Peptostreptococcaceae* showed a significant negative log fold change, confirming its depletion in women with RIF. This quantitative approach highlights the direction and extent of microbial shifts associated with infertility, strengthening the interpretation that gut dysbiosis in this cohort involves not only loss of beneficial taxa, but also enrichment in potentially harmful families.

The genus-level analysis of mean relative abundances between fertile women and women with RIF (Figure 6B) revealed significant compositional shifts in key microbial taxa. Among the genera depleted in women with RIF, *Phocaeicola*, formerly *Bacteroides vulgatus*, plays a role in the fermentation of complex polysaccharides [73,74], contributing to SCFA production and mucosal–metabolic homeostasis. *Anaerobutyricum*, *Anaerostipes*, and *Fusicatenibacter* are potent butyrate producers [75,76], supporting gut barrier integrity, and exert anti-inflammatory effects [77]. *Oscillibacter*, another genus reduced in women with RIF, has been implicated in cholesterol metabolism [78] and is known to interact with bile acid pathways and inflammation control [79]. Finally, the genus *Rombustia*, involved in the fermentation of simple carbohydrates and amino acids [80] and contributing to the local energy supply and pH regulation, is typically considered part of a healthy core microbiota. Although it is not a major butyrate producer, its bile salt hydrolase activity supports bile acid metabolism, which can influence lipid absorption, host–microbe signaling, and mucosal barrier integrity [81]. Moreover, *Romboutsia* species can form spores, increasing their resilience to environmental stress (e.g., dietary changes, antibiotics) [82].

In contrast, genera enriched in women with RIF include different pro-inflammatory or metabolically disruptive taxa. Among them, the genera *Bacteroides*, *Bilophila*, and *Gemmiger* appear to be significantly increased.

Although the genus *Bacteroides* is generally associated with a healthy core microbiota, due to the production of SCFAs such as acetate and propionate and its contribution to the synthesis of vitamins (e.g., B12, K) [77], its excessive abundance may exert detrimental effects. Indeed, Bacteroides species contribute to mucus layer degradation and to the production of lipopolysaccharides (LPS) with inflammatory activity, thereby compromising the intestinal barrier and increasing gut permeability [83]. Moreover, elevated *Bacteroides* abundance has been negatively associated with reproductive outcomes in some studies, especially when accompanied by reduced microbial diversity and depletion of butyrate-producing genera [66].

*Bilophila*, a sulfite-reducing bacterium, has been directly implicated in hydrogen sulfide-mediated mucosal damage and increased intestinal permeability [84]. Its expansion in the RIF group is consistent with increased inflammation and barrier dysfunction. Finally, *Gemmiger* is already associated with reproductive disorders [72].

Also, at the genus level, to quantify the magnitude of the observed taxonomic differences, a log fold change analysis was conducted, where only taxa with statistically significant differences were retained (Figure 8). The analysis confirmed the observed depletion in the RIF cohort of *Phocaeicola*, *Anaerobutyricum*, *Oscillibacter*, *Rombustia Anaerostipes*, and *Fusicatenibacter* and the enrichment of *Biophila*, *Gemmiger*, *Lachnoclostridium*, *Paraeggerthella*, and *Subdoligranulum*.

Overall, this microbial pattern reflects a dual phenomenon: a marked loss of beneficial SCFA producers critical for mucosal and systemic homeostasis and a gain of taxa associated with inflammation, bile acid dysregulation, and barrier impairment [77]. The depletion of genera such as *Anaerobutyricum*, *Phocaeicola*, and *Oscillibacter* may reduce epithelial tight junction stability, impair T-regulatory cell induction, and increase gut-derived endotoxemia [75,85,86]. Simultaneously, enrichment of *Bilophila* and other sulphide-producing microbes may exacerbate mucosal injury through hydrogen sulphide-mediated degradation of the mucus layer [79,84]. In addition, the rise of bile acid-modulating genera such as *Paraeggerthella* and *Bilophila* suggests interference with enterohepatic signaling pathways, which have been recognized as regulators of steroid metabolism and fertility [87,88].

Overall, the differences shown at both family and genus levels between fertile women and women with RIF support the hypothesis that infertility is associated with (i) reduced microbial-derived metabolic activity, particularly SCFA production; (ii) increased pro-inflammatory taxa; (iii) mucosal dysregulation; and (iv) microbial-driven immune activation, all hallmarks of gut–reproductive axis integrity and contributing to the maintenance of immune and inflammatory homeostasis.

Finally, the heatmap (Figure 9) provides an immediate overview of genus-level microbial distribution, offering a comprehensive profile beyond phylum- or family-specific analyses. While the first section of the heatmap appears to be relatively conserved across groups, the subsequent two sections reveal contrasting abundance patterns between fertile and infertile women. Although some overlap between groups is observed, infertile patients generally tend to share more similar microbial configurations, which differ from those commonly observed in fertile controls. Overall, the heatmap suggests a tendency toward group-associated microbial clustering, supporting the presence of community-level compositional differences rather than isolated taxonomic variations.

This study presents several strengths with respect to previously published work. Most notably, it focuses on the GM of infertile patients, an area that remains underexplored compared to the vaginal and endometrial microbiota. By including a well-characterized control group of women who were proven to be fertile, our study provides a robust comparative framework that enhances the biological relevance of the findings. Furthermore, the analysis was conducted at multiple taxonomic levels, including OTU, phylum, family, and genus levels, and incorporated alpha and beta diversity measures, Gram staining classification, and the Firmicutes/Bacteroidetes ratio. This comprehensive approach allowed for a deeper understanding of microbial differences associated with reproductive health.

However, the study also has relevant limitations: The first one is the selection of naturally fertile pregnant women as controls rather than infertility-matched women or IVF patients with successful implantation. Therefore, the microbial differences observed should not be interpreted as a gut microbiome signature specific to implantation failures. Rather, they may reflect a combination of factors related to infertility status, exposure to ART, reproductive history, metabolic and immune characteristics, and pregnancy-associated physiological changes, all of which are known to influence gut microbiome composition. The primary aim of this study was to explore gut microbiome differences between a well-defined infertile population with recurrent implantation failure and a fertile control group. Moreover, the absence of detailed metabolic and pharmacological data, such as data on metformin use, precludes adjustment for other potential confounders known to influence GM composition. While the sample size was sufficient to detect significant differences, a larger cohort would strengthen the statistical power and generalizability of the results. Finally, the lack of functional analysis, such as metagenomics or predictive pathway profiling, limits our ability to draw conclusions about the metabolic and immunological impact of the identified microbial shifts. Future studies including infertility-matched controls and IVF patients with successful implantation are warranted to better disentangle microbiome features associated with infertility from those potentially related to implantation failure itself.

## 5. Conclusions

In summary, our findings indicate that infertility is associated not only with an overall reduction in microbiota abundance, particularly the abundance of those microbes involved in SCFA metabolism, but also with specific compositional shifts toward pro-inflammatory bacterial families. This highlights a microbial pattern that may negatively impact reproductive competence through chronic low-grade inflammation, impaired gut barrier function and immune–endocrine crosstalk dysregulation. Despite limitations, our findings underline the importance of considering GM composition in infertile patients, focusing on the population with RIF. Indeed, the GM represents an actionable factor, as it can be modulated within a relatively short time frame through targeted dietary interventions, probiotic supplementation, or other lifestyle modifications. Its abundance and the possibility of collecting samples in a non-invasive manner make it an accessible and highly informative biomarker. These features render the GM a unique and promising target for patients facing implantation-related challenges, which remain the greatest “black box” in IVF, particularly in cases where euploid blastocysts are transferred without achieving pregnancy [5].

Future studies should be performed through longitudinal or interventional designs, integration of dietary and metabolic profiling, and incorporation of functional metagenomic analyses to better elucidate the role of the gut microbiota in implantation success.

## Figures and Tables

**Table 1:** Baseline characteristics of the study population. Values are expressed as mean and Standard Deviation (M ± SD).

Variable	Total	Control	RIF
Age (years), mean ± SD	38.46 ± 4.54	38.71 ± 4.52	38.2 ± 4.66
Years of infertility, mean ± SD	-	-	3.9 ± 2.3
Height (cm), mean ± SD	165.5 ± 5.98	164.6 ± 7.01	166.45 ± 4.67
Weight (kg), mean ± SD	61.98 ± 9.9	62.37 ± 10.49	61.56 ± 9.49
BMI (kg/m^2^), mean ± SD	22.58 ± 3.05	22.93 ± 2.69	22.21 ± 3.18
Previously transferred blastocysts that failed to implant, mean ± SD			2.5 ± 0.8, range 2–5
Own untested			0.8 ± 1.4, range 0–5
Donor untested			0.8 ± 1.2, range 0–3
Euploid			1.0 ± 1.1, range 0–3
Interval between stool sample collection and unsuccessful embryo transfer (months).			5.4 ± 3.1, range 2–12

Abbreviations: BMI, body mass index.

**Figure A1:**
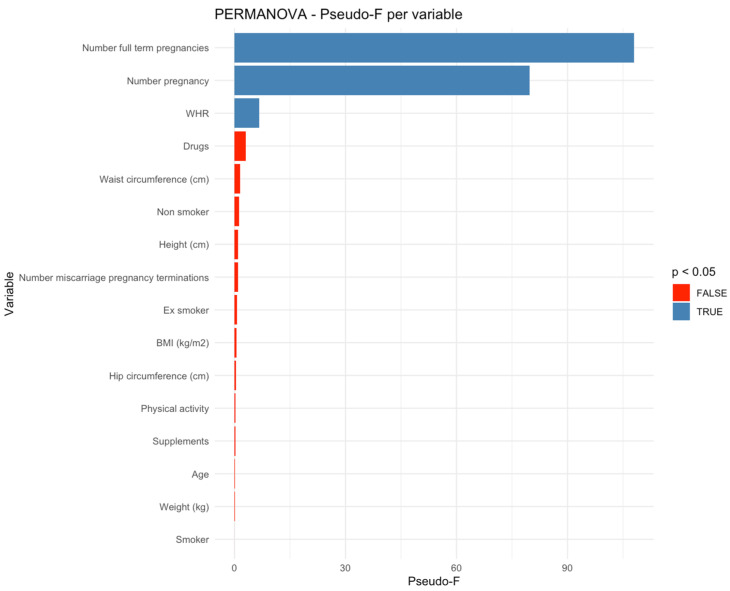
Baseline variables evaluated using permutational multivariate analysis of variance (PERMANOVA). PERMANOVA (permutational multivariate analysis of variance) was applied to assess the contribution of baseline clinical and demographic variables to inter-individual variability prior to microbiota analysis. Variables are shown on the *y*-axis and ordered by decreasing pseudo-F statistic, which represents the ratio of between-group to within-group variance. Statistical significance was defined as *p* < 0.05. WHR, waist-to-hip ratio; R^2^, proportion of variance explained by the variable.

## Data Availability

The data presented in this study are available on request from the corresponding author due to privacy issues.

## Abbreviations

The following abbreviations are used in this manuscript:

ANCOM-BC	Analysis Of Compositions Of Microbiomes With Bias Correction
ART	Assisted Reproduction Technologies
BMI	Body Mass Index
CVDs	Cardiovascular Diseases
ESHRE	European Society Of Human Reproduction And Embryology
F/B	Firmicutes-To-Bacteroidetes Ratio
GM	Gut Microbiota
GUSB	Β-Glucuronidase
HIV/AIDS	Human Immunodeficiency Virus/Acquired Immunodeficiency Syndrome
IBD	Inflammatory Bowel Disease
IVF	In Vitro Fertilization
logFC	Log Fold Change
LPS	Lipopolysaccharide
NGS	Next-Generation Sequencing
OTU(s)	Operational Taxonomic Unit (S)
PCoA	Principal Coordinates Analysis
PCOS	Polycystic Ovary Syndrome
PERMANOVA	Permutational Multivariate Analysis Of Variance
QIIME	Quantitative Insights Into Microbial Ecology
R^2^	Coefficient Of Determination (Proportion Of Variance Explained)
RIF	Recurrent Implantation Failure
SCFA(s)	Short-Chain Fatty Acid(S)
SD	Standard Deviation
TLR4	Toll-Like Receptor 4
UFs	Uterine Fibroids
WHO	World Health Organization
WHR	Waist-To-Hip Ratio
p_adj/padj	Adjusted *p*-Value
